# A multifactorial framework of psychobehavioral determinants of coping behaviors: an online survey at the early stage of the COVID-19 pandemic

**DOI:** 10.3389/fpsyt.2023.1200473

**Published:** 2023-08-10

**Authors:** Yi Ding, Ryo Ishibashi, Tsuneyuki Abe, Akio Honda, Motoaki Sugiura

**Affiliations:** ^1^Institute of Development, Aging and Cancer, Tohoku University, Sendai, Japan; ^2^Japan Society for the Promotion of Science, Tokyo, Japan; ^3^Center for Information and Neural Networks (CiNet), Osaka, Japan; ^4^Graduate School of Arts and Letters, Tohoku University, Sendai, Japan; ^5^Faculty of Informatics, Shizuoka Institute of Science and Technology, Fukuroi, Japan; ^6^International Research Institute of Disaster Science, Tohoku University, Sendai, Japan

**Keywords:** COVID-19, coping behavior, risk perception, psychobehavioral characteristic, model

## Abstract

Coronavirus disease 2019 dramatically changed people’s behavior because of the need to adhere to infection prevention and to overcome general adversity resulting from the implementation of infection prevention measures. However, coping behavior has not been fully distinguished from risk perception, and a comprehensive picture of demographic, risk-perception, and psychobehavioral factors that influence the major coping-behavior factors remain to be elucidated. In this study, we recruited 2,885 Japanese participants. Major coping-behavior and risk-perception factors were identified via exploratory factor analysis of 50 candidate items. Then, we conducted a hierarchical multiple regression analysis to investigate factors associated with each coping-behavior factor. We identified four types of coping behavior [CB1 (mask-wearing), CB2 (information-seeking), CB3 (resistance to social stagnation), and CB4 (infection-prevention)] and three risk-perception factors [RP1 (shortages of daily necessities), RP2 (medical concerns), and RP3 (socioeconomic concerns)]. CB1 was positively associated with female sex and etiquette. CB2 was positively related to RP1 and RP3. CB3 was positively related to RP1 and leadership, and negatively associated with etiquette. CB4 was positively associated with female sex, etiquette, and active well-being. This parsimonious model may help to elucidate essential social dynamics and provide a theoretical framework for coping behavior during a pandemic.

## 1. Introduction

Since the first case of coronavirus disease 2019 (COVID-19) was reported to the WHO, governments have spared no effort to prevent infection and transmission of this virus (1). As a health disaster (2), the response to this novel infectious disease is a public health issue that has had psychological and behavioral effects on individuals. Many studies have reported severe mental-health problems related to COVID-19, such as anxiety, depression, and suicide (3). People wore masks, disinfected their hands, and avoided crowds to prevent infection and transmission of the virus. Simultaneously, they had to manage the social disruption caused by the pandemic and precautions, such as shortages of masks and toilet paper and losses of jobs and important events. In such a stressful environment, it is vital to understand how individuals managed adversity (i.e., both infection and general adversity) to try to maintain their current standard of living.

Recent studies have found positive associations between two types of coping behaviors (infection prevention and general-adversity coping behaviors) and risk perception. For example, self-isolation was found to be positively associated with risk perception of personal safety and health services (4). Information seeking was positively associated with perceived risk at the individual and community levels (5), while behavioral change (e.g., informing others about COVID-19) was positively associated with the perceived risk of infection (6, 7). This effect of risk perception on coping behavior is consistent with findings for other types of hazards, such as hurricanes, tornadoes, earthquakes, and volcanoes (8–13).

Psychobehavioral characteristics have been analyzed in recent studies of COVID-19 coping behavior. Recent COVID-19-related studies have extensively used the Big Five scale to explore the psychological mechanisms underlying coping behavior, as the characteristics in this scale have profound implications for public health (14). Their findings suggest that the Big Five personality traits are significantly associated with infection preventive behaviors (15–24). For example, extraversion, openness, conscientiousness, and agreeableness are positively associated with infection preventive behaviors, whereas neuroticism is negatively associated with such behaviors (15, 21).

However, a consensus regarding these findings is difficult to achieve due to the lack of a common model for the main coping-behavior and risk-perception factors, leading to labels that differ in their definition, level of specificity, or conceptual overlap in different studies. For example, coping behavior has been defined at different levels of specificity, such as infection prevention measures (15) but also as problem-focused and emotion-focused coping behaviors (25). Similar risk perception labels have been used to measure different phenomena. For example, risk perception has been used to represent the degree to which people perceive COVID-19 to be a dangerous disease (26) but also as a proxy for the level of knowledge of the risks associated with COVID-19 (5). Different labels have also been used to measure similar perceptions of risk, such as the likelihood of infection (21) and concerns related to COVID-19 (15). Furthermore, coping behavior and risk perception have not been fully distinguished. Researchers have attempted to identify risk-perception factors from risk-related items rather than from a mixed pool containing coping behaviors (15).

In addition, current studies have not investigated the contribution of survival-oriented psychobehavioral characteristics to coping behaviors, which may provide more nuanced insights into individual differences in the perceptions of and responses to pandemics as a health disaster. We are interested in the Power to Live scale, which was developed in the context of the 2011 Tohoku earthquake and tsunami. This scale assesses eight psychobehavioral characteristics that are advantageous to survival: leadership, problem-solving, altruism, stubbornness, etiquette, emotional regulation, self-transcendence, and active well-being. Compared to the Big Five scale, the Power to Live scale provides a more nuanced understanding of the relationships between psychobehavioral characteristics and coping behaviors, particularly in the context of disasters (12, 27, 28).

We aimed to address two issues in this study. First, we identified major coping-behavior and risk-perception factors. Then, we examined important demographic, risk-perception, and psychobehavioral factors that contributed to coping behavior. We used a battery of questionnaires to investigate coping behaviors and risk perceptions in relation to COVID-19, as well as to obtain demographic information and measure psychobehavioral characteristics. We used the Power to Live scale based on our research interest and the Big Five scale according to previous studies (15–22). First, we conducted an exploratory factor analysis of various coping-behavior and risk-perception items to identify the major factors. Then, we examined the contributions of demographic, risk-perception (as an exploratory factor), and psychobehavioral characteristics to coping-behavior factors. We predicted that we would identify several coping-behavior and risk-perception factors associated with infection and general adversity. In addition to demographic and risk-perception factors, we hypothesized that associations would be found between coping-behavior factors and survival-oriented psychobehavioral characteristics.

## 2. Materials and methods

### 2.1. Participants

Data collection was conducted online by Neo Marketing (Tokyo, Japan) from March 19 to 24, 2020, during the early phase of the pandemic in Japan. The first coronavirus death had been reported (February 13); people had experienced the nationwide closure of elementary and junior high schools (March 2) and a national shortage of masks and toilet paper had occurred (March). The survey company emailed invitations to online crowdworkers living in all 47 prefectures of Japan. Participants were divided into six age groups (20s, 30s, 40s, 50s, 60s, and ≥70s) and two sex groups (male and female). Data were collected from 300 respondents in each sex and age group (total of 3,600 participants). All participants were required to have access to the Internet, to be familiar with working online, and to have sufficient time to fill in and submit the online questionnaire. Ultimately, we obtained data from 3,600 respondents (mean age = 49.73 ± 16.75 years). In addition to these 3,600 respondents, 481 participants were previously excluded due to inconsistencies between registered and reported demographic information or identical responses to all questions. We also excluded 715 satisfiers (i.e., people who presumably responded to the questions simply to meet the minimum requirements to finish the session, or people who responded carelessly) whose response time was <4 min (see the Supplemental material for further details regarding this criterion) resulting in a valid dataset of 2,885 individuals (1,524 women, mean age = 52.23 ± 16.52 years). No respondents had COVID-19.

### 2.2. Measures

The survey was developed in three sections (*n* = 104 in total), including five aspects of COVID-19-related items, consisting of coping behavior (*n* = 33), risk perception (*n* = 17), demographic questions (*n* = 10), and two psychobehavioral scales (*n* = 44).

#### 2.2.1. Coping behavior and risk perception

We used 50 items to measure coping behavior and risk perception. These items were taken from previous studies or generated from interviews with people in our network (29, 30). We assessed five aspects of coping behavior and risk perception: self-infection, other-infection, daily shortages of necessities, social and economic impacts, and information access (see Supplemental material).

#### 2.2.2. Demographic information

The demographic questions included 10 items (Table 1): sex, age, place of residence, family structure, have toddlers or not, have children or not, reported local cases of infection, the degree of risk of self-infection becoming severe because of chronic disease or age (two separate items), and the degree of risk of severe disease among their family members because of underlying disease or age.

**Table 1:** Demographic data of the participants.

Item		*N*
Sex	Male	1,361 (47%)
	Female	1,524 (53%)
Age	20s	364 (13%)
	30s	423 (15%)
	40s	470 (16%)
	50s	500 (17%)
	60s	548 (19%)
	≥70s	580 (20%)
Household structure	Single	525 (18.2%)
	Couple	888 (30.9%)
	Two generations (parents and children)	1,232 (42.8%)
	Three generations (parents, children, and grandchildren)	179 (6.1%)
	Other	61 (2.1%)
Toddlers in the household	Yes	250 (9%)
	No	2,635 (91%)
Children in the household	Yes	322 (11%)
	No	2,563 (89%)
Local cases of infection	Yes	1,109 (38%)
	No (including “*Do not know*” responses)	1776 (62%)	Knowledge
	1 (I have no expertise or experience with infectious diseases)	1,043 (36%)
	2	597 (21%)	3	582 (20%)
4	458 (16%)
5	162 (6%)
6 (I have extensive expertise and experience with infectious diseases)	43 (1%)	Chronic disease
1 (I do not have a chronic disease that can cause severe infection)	1,666 (58%)
2	437 (15%)	3	194 (7%)
4	228 (8%)
5	173 (6%)
6 (I have chronic diseases that can cause severe infection)	187 (6%)	High-risk age
1 (At my age, infection is unlikely to be severe)	1,021 (35%)
2	457 (16%)	3	367 (13%)
4	417 (14%)
5	344 (12%)
6 (At my age, infection is likely to be severe)	279 (10%)
High-risk family members	1 (My family members are unlikely to develop severe infection because of chronic disease or age)	1,139 (39%)
	2	330 (11%)
	3	272 (9%)
	4	334 (12%)
	5	332 (11%)
	6 (My family members are likely to develop severe infection because of chronic disease or age)	478 (17%)

Ordinal variables (e.g., age) were coded according to degree. Binary variables were coded as 1 or 0 [i.e., sex (male = 1; female = 0) and yes/no items (“Yes” = 1, “No = 0”)]. Household structure was coded as a dummy variable.

#### 2.2.3. Psychobehavioral characteristics

We used the 34-item Power to Live scale, which measures eight psychobehavioral characteristics: leadership, problem-solving, altruism, stubbornness, etiquette, emotional regulation, self-transcendence, and active well-being. Previous studies have demonstrated internal consistency and concurrent validity of the scale (31, 32). Participants provided responses using a six-point scale (0: *Not at all*; 5: *Very much*). We calculated the mean score for each characteristic.

We also assessed the Big Five personality traits using the Japanese version of the Ten-Item Personality Inventory (TIPI-J). The TIPI-J has good internal consistency and concurrent validity (33, 34). Participants provided responses using a six-point scale (0: *Not at all*; 5: *Very much*). Each of the five dimensions (extraversion, agreeableness, conscientiousness, neuroticism, and openness) included a positive and a reverse item. Dimension scores were calculated by subtracting the score for the reverse item from that for the positive item.

## 3. Analysis

All analyses were performed in R (35) using the tidyverse (36), psych (37), GPArotation (38), EFA.MRFA (39), parameters (40), and effectsize (41) packages.

### 3.1. Exploratory factor analysis

We performed a factor analysis by pooling all of the coping-behavior and risk-perception items. The aim of the factor analysis was to dissociate coping behavior and risk perception by eliminating items that may convey similar nuances of both. First, we confirmed the appropriateness of the data for exploratory factor analysis by performing the Kaiser–Meyer–Olkin (KMO) test and Bartlett’s test (42). The number of factors was determined based on the minimum average partial procedure (43), the Hull method (44), a parallel analysis (45), and a scree plot. We used the maximum likelihood method with Promax rotation because we assumed that the identified factors were correlated, and this method is well suited to simple structures (46). We excluded items if they met any of the following criteria: commonality <0.3, loading <0.4, or loading >0.4 on more than one factor (cross-loading). After removing an item, we repeated the analysis until all items met the criteria. The sum of squared (SS) loadings indicated the proportion of the variance explained by each factor. Cronbach’s *α* was calculated for each factor to estimate internal consistency. Factor scores were calculated by averaging the scores of all items for each factor.

### 3.2. Correlation analysis

We performed a correlation analysis to explore the relationships among the identified coping-behavior factors, risk-perception factors, and psychobehavioral characteristics. Given the large sample size, we used |*r*| > 0.3 as the effect size threshold (47, 48).

### 3.3. Hierarchical regression analysis

To further explore the factors contributing to coping behavior and risk perception, we performed hierarchical regression analysis, which provides significant tests for the effects of independent variables on the dependent variable while controlling for the influence of the other independent variables.

We performed hierarchical regression analyses for each of the four coping-behavior factors. With the factor score as the dependent variable, we used 13 background factors in the first block, 3 risk-perception factors in the second block, and 13 psychobehavioral characteristics in the third block as explanatory variables.

We applied similar hierarchical regression models for each of the three risk-perception factors. We entered the 13 background-factor variables in the first block and the 13 psychobehavioral characteristics in the second block as explanatory variables.

Hierarchical regression analysis was performed using the stepwise method, and the variables were selected based on Akaike’s information criterion. For each regression model, we calculated tolerance and the variance inflation factor to detect multicollinearity among predictors. Tolerance values <0.2 and variance inflation factor values >4 are considered problematic (49). Cohen’s *f*^2^ was used reflect the overall effect size of each block in the hierarchical regression (47). The term *f*_B/A_^2^ represents the effect size of each predictor (50). Due to the large sample size, we used a small effect size (i.e., Cohen’s *f*^2^ = 0.02) as the threshold.

## 4. Results

The demographic data are summarized in Table 1. The numbers and percentages of participants’ demographic information are given for each item.

### 4.1. Exploratory factor analysis

The results of the KMO and Bartlett’s tests indicated that the data were suitable for factor analysis (KMO = 0.92; *χ*^2^ (1225) = 75783.41, *p* < 0.001). The minimum average partial, Hull method, and scree plot suggested extraction of eight factors, while the parallel analysis suggested extraction of 11 factors. Therefore, we selected an eight-factor solution; however, the results had a factor containing only two items. Thus, we eliminated one factor and reached a seven-factor solution following the suggestion that a factor should include at least three items (51). Finally, we removed nine items based on the commonality criterion and three items based on the two loading criteria; we thus achieved satisfactory results for the seven-factor solution (Table 2).

**Table 2:** Factor analysis of the COVID-19 questionnaire.

Items	CB1	RP1	CB2	RP2	CB3	RP3	CB4	*α*
CB1: Mask-wearing								**0.89**
I wear a mask to avoid infecting others	**1.02**	−0.07	0.03	−0.01	−0.01	−0.01	−0.09	
I wear a mask so that people around me do not feel uncomfortable if I cough or sneeze	**0.98**	−0.05	−0.01	−0.04	−0.02	0.01	−0.03	
I wear a mask to prevent myself from becoming infected	**0.71**	0.01	−0.02	0.01	0.06	−0.08	0.14	
I cover my mouth and nose when I cough or sneeze to avoid infecting others	**0.47**	0.04	−0.02	0.00	−0.11	0.15	0.23	
RP1: Shortages of daily necessities								**0.79**
I am worried about shortages of daily necessities caused by disruptions in production and distribution related to the spread of infection	−0.04	**0.99**	0.01	−0.05	−0.10	−0.01	0.07	
I am worried that daily necessities may not be sufficient because of hoarding	−0.04	**0.92**	−0.03	−0.03	−0.08	−0.03	0.08	
I am worried that lifelines (water, gas, electricity) may be cut off because of the social chaos caused by the spread of infection	0.00	**0.44**	0.01	0.08	0.25	0.00	−0.12	
CB2: Information-seeking								**0.92**
I frequently check the national and local governments’ responses to the COVID-19 pandemic and predictions about what will happen	−0.02	−0.02	**0.93**	−0.01	−0.13	0.01	0.05	
I frequently check on the number of people infected with the coronavirus	0.02	0.01	**0.91**	−0.01	−0.14	−0.02	−0.01	
I frequently check on the social and economic impacts of infection control	−0.04	−0.02	**0.82**	0.01	−0.04	0.08	0.06	
I frequently check on the shortages of daily necessities	0.02	0.13	**0.71**	−0.03	0.11	−0.07	−0.02	
I collect information from specialized organizations, such as the Ministry of Health, Labor, and Welfare	0.00	−0.11	**0.70**	0.03	0.20	−0.05	0.02	
I monitor COVID-19-related news on TV and in the newspapers	0.01	0.00	**0.68**	−0.05	−0.12	0.15	0.06	
I spend time searching for COVID-19-related information on the internet	−0.01	0.01	**0.67**	0.03	0.16	−0.01	−0.08	
RP2: Medical concerns								**0.87**
I am worried that I will become infected	−0.04	−0.10	−0.01	**0.91**	−0.03	−0.02	0.09	
I am worried that my family and friends will become infected	−0.07	−0.05	−0.05	**0.85**	−0.08	−0.00	0.11	
I am worried that I will be infected and it will be serious	0.00	−0.11	0.08	**0.74**	−0.04	−0.08	0.07	
I am worried that many people around us will become infected	−0.06	−0.06	−0.04	**0.68**	0.10	−0.01	−0.01	
I am worried about infecting others (if I were infected)	0.06	−0.01	−0.01	**0.67**	0.06	−0.01	−0.07	
I am worried that people around me may think that I am infected and feel anxious when I cough	0.07	0.07	0.00	**0.56**	0.06	0.01	−0.04	
When I or my family members are infected, I am worried that I will not be able to respond appropriately	0.01	0.08	−0.04	**0.53**	−0.07	0.06	−0.06	
When I or my family are infected, I am worried that the medical system will not be adequate to manage the infection	−0.01	0.17	−0.01	**0.48**	−0.13	0.10	0.03	
CB3: Resistance to social stagnation								**0.80**
I get together with my friends and relatives to stay in touch, particularly during these times	−0.02	−0.01	−0.05	−0.06	**0.76**	0.00	0.06	
I try to spend my money, particularly during these times	−0.01	−0.10	−0.08	0.03	**0.69**	0.00	−0.01	
I communicate using the phone, email, or text, particularly during these times	−0.01	−0.03	0.03	−0.04	**0.69**	0.03	0.12	
I try to do fun things, particularly during these times	−0.05	−0.03	−0.11	−0.08	**0.65**	0.14	0.13	
I try to advise my friends and acquaintances not to buy extra things that they do not need right now	−0.02	0.05	0.06	−0.01	**0.54**	−0.1	0.07	
I advise my friends and acquaintances to stockpile daily necessities	0.10	0.07	0.11	0.06	**0.51**	−0.15	−0.05	
I am worried that refraining from events will lead me to lose events that are important in my life	0.01	0.01	0.03	0.04	**0.44**	0.26	−0.16	
RP3: Socioeconomic concerns								**0.83**
I am worried that the spread of infection will not be under control after April and that this situation will continue	0.00	−0.09	0.01	0.02	−0.06	**0.92**	−0.01	
I am worried that the situation may worsen in the future, causing further turmoil in society	0.00	−0.07	0.01	0.07	0.07	**0.85**	−0.09	
I am worried that economic stagnation could affect many people because of poor corporate balance, bankruptcy, and job loss.	0.00	0.09	0.07	−0.03	−0.10	**0.61**	0.11	
I am worried that refraining from events will lead many people to lose events that are important in their lives	0.01	0.03	0.00	−0.03	0.12	**0.56**	0.04	
CB4: Infection-prevention								**0.81**
I ventilate rooms to prevent infection	−0.04	0.07	0.00	0.00	0.16	−0.08	**0.72**	
I have been washing my hands well and gargling regularly	0.11	0.09	−0.09	0.01	0.02	0.05	**0.66**	
I avoid going to crowded places	−0.04	0.01	0.05	0.02	−0.10	0.06	**0.65**	
I am careful about physical condition management, such as eating, exercising, and sleeping	−0.05	−0.04	0.07	−0.01	0.07	0.03	**0.63**	
I try not to touch door handles or buttons that are touched by many people unknown to me	0.07	−0.04	0.04	0.08	0.14	−0.07	**0.59**	
SS loadings	2.79	2.18	4.34	3.89	3.01	2.47	2.37	
Cumulative variance	0.07	0.13	0.24	0.35	0.43	0.49	0.55	

Loadings ≥ 0.40 are in bold. *α*: Cronbach’s alpha.

Table 2 shows the results of the seven factors. There were four coping-behavior factors: two related to infection, CB1, mask-wearing (representing mask use related to infection prevention behavior); and CB4, infection-prevention (representing general infection prevention measures, such as hand washing); and two related to general adversity, CB2, information-seeking (searching for or checking COVID-19-related information) and CB3, resistance to social stagnation. The CB3 label was based on the fact that all described behaviors serve to prevent social stagnation. This stagnation may be caused by reduced communication, reduced economic activity, and psychological depression. Items 5 and 6 are also behaviors that counteract social disorders, albeit from different viewpoints. There were three risk-perception factors: one related to infection, RP2, medical concerns (indicated concerns about medical resources and becoming infected); and two related to general adversity, RP1, shortages of daily necessities (measured concerns about shortages of daily supplies) and RP3, socioeconomic concerns (represented concerns about society and the economy). The internal consistency coefficients (Cronbach’s *α*) of all factors were >0.70. They constituted 55% of the total variance.

### 4.2. Correlation analysis

Among the coping-behavior factors, information-seeking (CB2) was significantly associated with all other coping-behavior factors (CB1, CB3, and CB4) and socioeconomic concerns (RP3). Furthermore, mask-wearing (CB1) was associated with infection-prevention (CB4). Three risk-perception factors were significantly associated with each other; socioeconomic concerns (RP3) were significantly associated with mask-wearing (CB1) and information-seeking (CB2) (Table 3).

**Table 3:** Correlation matrix for coping-behavior factors, risk-perception factors, and psychobehavioral characteristics.

		Coping behaviors				Risk perceptions		
		CB1	CB4	CB3	CB2	RP2	RP1	RP3
CB1	Mask-wearing	—						
CB4	Infection-prevention	0.574*	—					
CB3	Resistance to social stagnation	0.135*	0.228*	—				
CB2	Information-seeking	0.374*	0.467*	0.380*	—			
RP2	Medical concerns	0.230*	0.159*	0.137*	0.237*	—		
RP1	Shortages of daily necessities	0.250*	0.180*	0.236*	0.279*	0.419*	—	
RP3	Socioeconomic concerns	0.309*	0.259*	0.170*	0.347*	0.340*	0.457*	—
	Power to Live							
	Leadership	0.152*	0.299*	0.468*	0.355*	−0.043	0.017	0.072*
	Problem-solving	0.229*	0.367*	0.217*	0.304*	−0.020	0.070*	0.209*
	Altruism	0.188*	0.217*	0.311*	0.301*	0.068*	0.104*	0.155*
	Stubbornness	0.088*	0.135*	0.171*	0.182*	0.049	0.101*	0.147*
	Etiquette	0.355*	0.437*	0.094*	0.315*	0.021	0.083*	0.255*
	Emotional regulation	0.186*	0.353*	0.256*	0.293*	−0.041	0.029	0.153*
	Self-transcendence	0.254*	0.368*	0.270*	0.349*	0.030	0.078*	0.196*
	Active well-being	0.200*	0.391*	0.342*	0.354*	−0.024	0.043	0.124*
	Big Five							
	Extraversion	0.055	0.131*	0.248*	0.161*	−0.102*	−0.064*	−0.011
	Agreeableness	0.217*	0.244*	0.007	0.178*	−0.108*	−0.043	0.102*
	Conscientiousness	0.126*	0.296*	0.073*	0.195*	−0.155*	−0.108*	−0.011
	Neuroticism	−0.025	−0.165*	−0.094*	−0.091*	0.210*	0.164*	0.079*
	Openness	0.004	0.126*	0.269*	0.157*	−0.050	−0.017	−0.015

|*r*| > 0.3 are in bold. *: *p*-values < 0.001.

All four coping-behavior factors were significantly associated with at least one characteristic in the Power to Live scale, but the risk-perception factors did not demonstrate such associations. Both infection prevention factors (CB1 and CB4) were associated with etiquette; infection-prevention (CB4) was additionally associated with problem-solving, emotional regulation, self-transcendence, and active well-being. Both general-adversity coping behaviors (CB2 and CB3) were associated with leadership, altruism, and active well-being; information-seeking (CB2) was additionally associated with problem-solving, etiquette, and self-transcendence. However, no significant associations were observed between factors and Big Five characteristics (Table 3).

### 4.3. Hierarchical regression analysis

Tolerance and variance inflation factor analyses indicated no evidence of multicollinearity in any hierarchical regression.

Table 4 summarizes the results of the four coping-behavior factors (see Supplementary Tables S1–S4 online). Among the demographic factors, sex negatively contributed to two infection prevention factors (CB1 and CB4). Among risk-perception factors, shortages of daily necessities (RP1) significantly contributed to two general-adversity coping behaviors (CB2 and CB3), while socioeconomic concerns (RP3) positively contributed to information-seeking (CB2). However, medical concerns (RP2) did not contribute to any of the coping behaviors. Among the psychobehavioral characteristics, etiquette was positively associated with two infection prevention factors (CB1 and CB4), while it was negatively associated with behaviors protecting against social stagnation (CB3). Leadership and active well-being were positively associated with resistance to social stagnation (CB3) and infection-prevention (CB4), respectively.

**Table 4:** Hierarchical regression analysis of coping-behavior factors.

	Mask-wearing		Infection-prevention		Resistance to social stagnation		Information-seeking	
	*β*	*f*_*B*/*A*_^2^	*β*	*f*_*B*/*A*_^2^	*β*	*f*_*B*/*A*_^2^	*β*	*f*_*B*/*A*_^2^
Sex	−0.232*	**0.059**	−0.179*	**0.035**	−0.026	0.001	−0.061*	0.004
Knowledge	0.078*	0.007	0.143*	**0.022**	0.176*	**0.032**	0.155*	**0.026**
Local case of infection	0.067*	0.005	0.084*	0.008			0.079*	0.007
Hs_single	−0.037	0.001	−0.027	0.001			−0.066*	0.005
High-risk age	0.167*	**0.030**	0.120*	0.010			0.156*	0.016
Toddler			0.053	0.003	0.033	0.001	0.036	0.001
Age			0.086*	0.005			0.110*	0.008
Child					0.057	0.003		
Hs_couple			0.035	0.001				
Block 1		0.106		0.119		0.037		0.119
∆*R*^2^		0.096		0.106		0.036		0.106
∆*F*		61.150*		42.759*		26.994*		48.733*
Medical concerns	0.068*	0.004	0.041	0.002			0.074*	0.005
Shortages of daily necessities	0.109*	0.010	0.084*	0.006	0.200*	**0.035**	0.159*	**0.023**
Socioeconomic concerns	0.209*	**0.041**	0.19*	**0.033**	0.072*	0.005	0.237*	**0.056**
+Block 2		0.116		0.076		0.064		0.175
∆*R*^2^		0.094		0.063		0.058		0.133
∆*F*		111.065*		72.681*		92.179*		167.796*
Leadership					0.483*	**0.118**	0.178*	0.015
Problem-solving					−0.177*	0.014	−0.076	0.003
Altruism	−0.042	0.002	−0.083*	0.006	0.101*	0.009	0.043	0.002
Stubbornness			−0.061	0.004	−0.052	0.003		
Etiquette	0.194*	**0.023**	0.181*	**0.022**	−0.250*	**0.043**		
Emotional regulation	−0.043	0.001	0.056	0.002	0.084*	0.004		
Self-transcendence	0.053	0.001	0.042	0.002	0.047	0.001	0.057	0.002
Active well-being	0.058	0.002	0.191*	**0.027**	0.163*	0.018	0.154*	0.016
Extraversion					−0.047	0.002	−0.035	0.001
Agreeableness	0.107*	0.011	0.034	0.002	−0.094*	0.009		
Conscientiousness			0.135*	0.019	−0.038	0.002	0.052	0.003
Openness	−0.035	0.001			0.103*	0.012		
+Block 3		0.087		0.222		0.429		0.130
∆*R*^2^		0.065		0.151		0.272		0.087
∆*F*		35.744*		79.676*		102.521*		53.093*

*: *p*-values < 0.001; *f*_B/A_^2^ > 0.02 are in bold; High-risk age: the age at which infection is likely to be severe. HS, household structure. Variables selected by the stepwise method are presented.

Table 5 displays a summary of the results for the risk-perception factors (see Supplementary Tables S5–S7 online). Among the demographic factors, age, high-risk age, and having a high-risk family member significantly contributed to medical concerns (RP2), but age demonstrated a negative association. Among psychobehavioral characteristics, etiquette contributed only to socioeconomic concerns (RP3). Table 6 shows the relationships of demographic information, risk-perception factors, and psychobehavioral characteristics with coping-behavior factors.

**Table 5:** Hierarchical regression analysis of risk-perception factors.

	Medical concerns		Shortages of daily necessities		Socioeconomic concerns	
	*β*	*f*_*B*/*A*_^2^	*β*	*f*_*B*/*A*_^2^	*β*	*f*_*B*/*A*_^2^
Age	−0.296*	**0.061**	−0.225*	**0.031**	−0.138*	0.011
High-risk age	0.351*	**0.054**	0.149*	0.011	0.197*	0.014
High-risk family member	0.177*	**0.029**	0.089*	0.006	0.073*	0.004
Sex			−0.048	0.002	−0.031	0.001
Child	0.030	0.001	0.042	0.002		
Local case of infection	0.039	0.002			0.048	0.002
Chronic disease	0.088*	0.006			−0.056	0.002
HS_two generations	0.040	0.002				
Knowledge					0.028	0.001
Block 1		0.249		0.052		0.037
∆*R*^2^		0.199		0.050		0.035
∆*F*		102.241*		30.109*		15.028*
Leadership					−0.102*	0.006
Problem-solving	0.171*	0.013	0.080	0.002	0.163*	0.010
Altruism	0.060	0.003	0.053	0.002		
Stubbornness			0.036	0.001	0.055	0.002
Etiquette	0.046	0.001	0.096*	0.004	0.213*	**0.023**
Emotional regulation	−0.061	0.002				
Self-transcendence			0.042	0.001	0.057	0.002
Extraversion	−0.076*	0.006	−0.069*	0.004		
Agreeableness	−0.100*	0.009	−0.048	0.002	0.053	0.002
Conscientiousness	−0.110*	0.010	−0.085*	0.005	−0.087*	0.006
Neuroticism	0.128*	0.013	0.141*	0.014	0.149*	0.017
Openness			0.032	0.001		
+Block 2		0.074		0.060		0.128
∆*R*^2^		0.055		0.054		0.109
∆*F*		26.601*		17.261*		45.795*

Other details are as shown in Table 4.

**Table 6:** Summary of the contributions of various factors to coping-behavior factors.

		Infection		General adversity	
		CB1 Mask-wearing	CB4 Infection-prevention	CB2 Information-seeking	CB3 Resistance to social stagnation
Demographic information	Female sex	+	+		
Risk-perception factors	RP1 Shortages of daily necessities			+	+
	RP3 Socioeconomic concerns			+	
Psychobehavioral characteristics	Leadership				+
	Etiquette	+	+		−
	Active well-being		+		

## 5. Discussion

The main goal of this study was to identify major coping-behavior factors while exploring the contributions of demographic information, risk-perception factors, and psychobehavioral characteristics to coping-behavior factors. We identified four coping-behavior factors (two related to infection and two related to general adversity) and three risk-perception factors (one related to infection and two related to general adversity). Female sex and etiquette promoted infection prevention behaviors (CB1 and CB4), whereas shortages of daily necessities (RP1) promoted general-adversity coping behaviors (CB2 and CB3). Active well-being promoted infection-prevention (CB4), and socioeconomic concerns (RP3) promoted information-seeking (CB2). Resistance to social stagnation (CB3) was inhibited by etiquette and promoted by leadership. Although some of the correlations between the Big Five scale and coping-behavior factors were consistent with previous studies (15, 21), none of them reached our effect-size threshold (Table 6).

The factors promoting infection prevention behaviors in this study were consistent with existing knowledge. Our findings showed that etiquette and female sex contributed to two infection prevention factors, while medical concerns (RP2) did not. Etiquette was defined as adherence to social norms (32), and infection prevention behaviors may arise from the desire to comply with social norms. Our finding that medical concerns (RP2) lacked an association with infection prevention behaviors while etiquette was associated with infection prevention behaviors was consistent with a previous Japanese study, in which mask-wearing was related to social norms rather than the perceived risk of COVID-19 (52). In addition, our finding that women were more inclined to exhibit infection prevention behaviors was consistent with studies in which women were more willing to self-isolate (53) and more frequently engaged in positive coping behavior than men (54).

There are two potential reasons for the identification of two infection prevention factors in this study (i.e., mask-related and mask-unrelated). The first involves the executability of the two types of infection prevention. The availability of masks may have caused a separation of infection prevention due to the severe shortage and hoarding of masks that occurred during the early stage of the pandemic (55). The second reason involves the emphasis on wearing masks. The Ministry of Health, Labor, and Welfare released 61 documents concerning the latest domestic coronavirus situation in 77 days [1/1/2020–3/18/2020 (immediately before the survey)]; each of the documents included the same message to the public that wearing masks and washing hands are important practices (56). Most indoor and public places required a mask before entering. Thus, people could be divided into two groups: a group that wore masks and followed the mask-wearing recommendation and a group that lacked masks and focused more on general infection prevention.

Shortage of daily necessities (RP1) is a common facilitative factor of general-adversity coping behaviors. Media-dependency theory claims that people become increasingly dependent on social media during severe social disruption (57). Consistent with this theory, our results reveal that individuals tended to use information-seeking strategies (CB2) to be informed, prepared, and responsive to COVID-19 when they knew about shortages of daily necessities. The contribution of shortages of daily necessities (RP1) to resistance to social stagnation (CB3) is congruent with the results of the disaster research described in the Introduction. Shortage of daily necessities, while not totally representing social stagnation, might be seen as an early warning sign of subsequent social stagnation in multiple fields, such as long-lasting impacts on the supply chain (58). Thus, when our participants perceived an existing threat to society (i.e., shortage of daily necessities), they may have responded as if social stagnation was imminent.

However, each of the two general-adversity coping-behavior factors had unique features. Except for shortages of daily necessities (RP1), information-seeking (CB2) was facilitated by socioeconomic concerns (RP3); resistance to social stagnation (CB3) was inhibited by etiquette and enhanced by leadership. The relationship between information-seeking (CB2) and socioeconomic concerns (RP3) is also consistent with the media-dependency theory: perceived social risk enhances information-seeking. The negative contribution of etiquette to resistance to social stagnation (CB3) may have originated from obedience to guidance. For instance, people with high etiquette scores are more likely to maintain social distance and limit their engagement in non-essential activities. On the other hand, people with strong etiquette skills might attempt to maintain their usual routines as before. They may not implement strategies to resist social stagnation because fussiness also violates social norms (59). The essence of leadership refers to the tendency to solve problems through communication, which may explain directly its contribution to responses to imminent social stagnation (CB3). An example item of leadership in the Power to Live scale is: “To resolve problems, I gather everyone involved together to discuss the matter.” People with strong leadership are more likely to take the initiative to reach out to others and solve problems. Previous studies have reported a contribution of leadership to spontaneous evacuation efforts in the context of an imminent tsunami (12), including encouraging other people to evacuate (27) and resolving problems through mutual aid (28).

Our study implies that each coping behavior has distinct facilitatory/inhibitory psychological processes supported by a partial conflict between survival-oriented characteristics. Etiquette facilitated two infection prevention factors (CB1 and CB4) but inhibited the general-adversity coping-behavior factor (i.e., CB3; resistance to social stagnation). This conflict may be the result of a trade-off between infection prevention and general-adversity coping behaviors. For example, maintaining social distance (or self-isolation) is an effective and critical method to stop transmission. However, people may not be able to attend important events or socialize, which makes them feel socially isolated and impairs both physical and mental health.

In summary, our results provide a theoretical framework for sorting out the apparently chaotic social responses to the pandemic into a comprehensive picture by identifying its major factors and investigating the psychobehavioral mechanism underlying each factor. Previous studies have addressed only the psychological factors involved in social responses to the COVID-19 pandemic (4, 5, 15, 21, 52, 60). Our findings imply that coping behaviors can be classified into two categories: infection prevention (CB1: mask-wearing and CB4: infection-prevention) and coping with general adversity (CB2: information-seeking and CB3: resistance to social stagnation). The former behaviors were associated with female sex and etiquette, and the latter behaviors were associated with concerns regarding shortages of daily necessities (RP1). Additionally, infection-prevention (CB4) was facilitated by active well-being. Information-seeking (CB2) was promoted by socioeconomic concern (RP3) and resistance to social stagnation (CB3) was facilitated by leadership and suppressed by etiquette. The opposite associations of etiquette between two infection prevention factors (CB1 and CB4) and resistance to social stagnation (CB3) may underlie trade-offs between these two types of coping behaviors; this perspective may become evident only in this comprehensive framework.

Our framework provides policymakers with a comprehensive picture of a public with different characteristics, the associated coping behaviors exhibited, and their contexts. This may help them to implement policies that maximize social benefits. For instance, to prevent overresponses to adversity, national and local governments can take steps to reduce concerns about shortages of daily necessities, such as by ensuring adequate supplies. It is important to note that increased concern can have unexpected consequences: increased concern about medical issues is unlikely to facilitate infection prevention behaviors, but increased socioeconomic concern may facilitate people’s information seeking and lead to an “infodemic” (61). To optimize the balance between infection prevention and resistance to social stagnation, governments should take age- and culture-specific psychobehavioral characteristics (i.e., leadership, etiquette, and active well-being) into account, or consider educational and intervention approaches to affect such psychobehavioral characteristics. Based on the current theoretical framework, the development of such a set of strategic social approaches to pandemics appears promising.

Our study had several limitations. First, our work may be preliminary with regard to building a comprehensive model; a truly comprehensive model would integrate results for multiple periods characterized by different social responses. It is necessary to consider survey results from other periods. However, we do not consider the current data to be less valuable than such results. Our data reflected the social situation in the early days of the pandemic when the features of COVID-19 were largely unknown, and people’s fears were at their highest. Social turmoil caused by the shortage of masks and toilet paper, for example, was also unique to this period. Second, the comprehensiveness of our results pertains only to individuals without COVID-19 infection, as none of our participants had COVID-19. Third, the sample is limited in its representativeness. Despite efforts to recruit participants from different generations from all over Japan, we acknowledge that the sample may not fully represent the larger population of interest. Finally, this study used self-report measures and included only Japanese participants, which may limit the generalizability of the findings. Moreover, there may have been response biases, such as population and optimism biases, where the participants were all familiar with online surveys and may have overestimated their knowledge of infectious diseases or underestimated the risk of disease. Such biases could have affected the relationships among the variables. Future studies could conduct experiments in other cultures and use other data-collection approaches to enhance the robustness and generalizability of the findings.

## 6. Conclusion

We proposed a new model comprising four independent coping-behavior factors and three risk-perception factors for COVID-19, which were categorized into infection-related and general adversity-related groups. We have demonstrated that infection prevention and coping with general adversity were associated with different factors. Female sex and etiquette promoted two infection prevention factors, while shortages of daily necessities promoted two general-adversity coping behavior factors. In addition, infection-prevention (CB4) was promoted by active well-being, and information-seeking (CB2) was promoted by socioeconomic concerns; meanwhile, resistance to social stagnation (CB3) was inhibited by etiquette and promoted by leadership. This study provides a theoretical framework for coping behaviors and risk perception during a pandemic and demonstrates their underlying psychobehavioral mechanisms. The contribution of demographic, risk-perception, and psychobehavioral characteristics to coping behavior could help policymakers devise effective strategies for optimizing social responses to pandemics. Future research should continue to refine this model of perceived risk and coping behavior, and including more data from different periods of the epidemic would greatly improve the model. Moreover, future studies can use this model to investigate how mental health and emotional distress affect different types of coping behavior.

## Data availability statement

The datasets presented in this study can be found in online repositories. The names of the repository/repositories and accession number(s) can be found at: https://osf.io/t4suw/.

## Ethics statement

The studies involving humans were approved by Ethical Committee of the International Research Institute of Disaster Science, Tohoku University (2019-035). The studies were conducted in accordance with the local legislation and institutional requirements. The participants provided their written informed consent to participate in this study.

## Author contributions

YD and MS designed the experiment. YD analyzed the data and wrote the initial draft of the manuscript. All authors contributed to the article and approved the submitted version.

## Funding

This study was supported by the Subsidy for Interdisciplinary Study and Research Concerning COVID-19 from the Mitsubishi Foundation, KAKENHI (22H04855 and 22KJ0310) from the Japan Society for the Promotion of Science, and JST SPRING (JPMJSP2114).

## Conflict of interest

The authors declare that the research was conducted in the absence of any commercial or financial relationships that could be construed as a potential conflict of interest.

## Publisher’s note

All claims expressed in this article are solely those of the authors and do not necessarily represent those of their affiliated organizations, or those of the publisher, the editors and the reviewers. Any product that may be evaluated in this article, or claim that may be made by its manufacturer, is not guaranteed or endorsed by the publisher.
