# Supplementary material for: A multifactorial framework of psychobehavioral determinants of coping behaviors: an online survey at the early stage of the COVID-19 pandemic
Source: Front Psychiatry. 2023 Aug 10;14:1200473. doi: 10.3389/fpsyt.2023.1200473 (PMC10448049; doi:10.3389/fpsyt.2023.1200473)
Supplement: Supplementary file 1 [file Data_Sheet_1.docx]

Supplementary Material

# A Multifactorial Framework of Psychobehavioral Determinants of Coping Behaviors: An Online Survey at the Early Stage of the COVID-19 Pandemic

Yi Ding^1,2 *^, Ryo Ishibashi^1,3^, Tsuneyuki Abe^4^, Akio Honda^5^, Motoaki Sugiura^1,6^

*** Correspondence:** Yi Ding (email: dingyi9508@outlook.com)

**Methods**

**Participants**

Poor data quality is inherent in online surveys. Low-quality data can affect the relationships between variables (1). There have been recommendations to exclude participants with extremely fast response times and inconsistent responses (2). Thus, we used cut-offs based on response time and response consistency to exclude some of the participants from our analysis. Response consistency was sensitively reflected in the correlations between reverse items in each dimension of the Big Five scale.

We calculated the moving average of the correlation between the two reverse items in each dimension of the Big Five scale. We found the correlation coefficients were negative when the reaction time exceeded 4 min (Figure S1).

**Figure S1.** Moving average correlation coefficients for each dimension in the Big Five scale. Vertical dashed line indicates a response time of 4 min and horizontal line indicates a coefficient of 0.


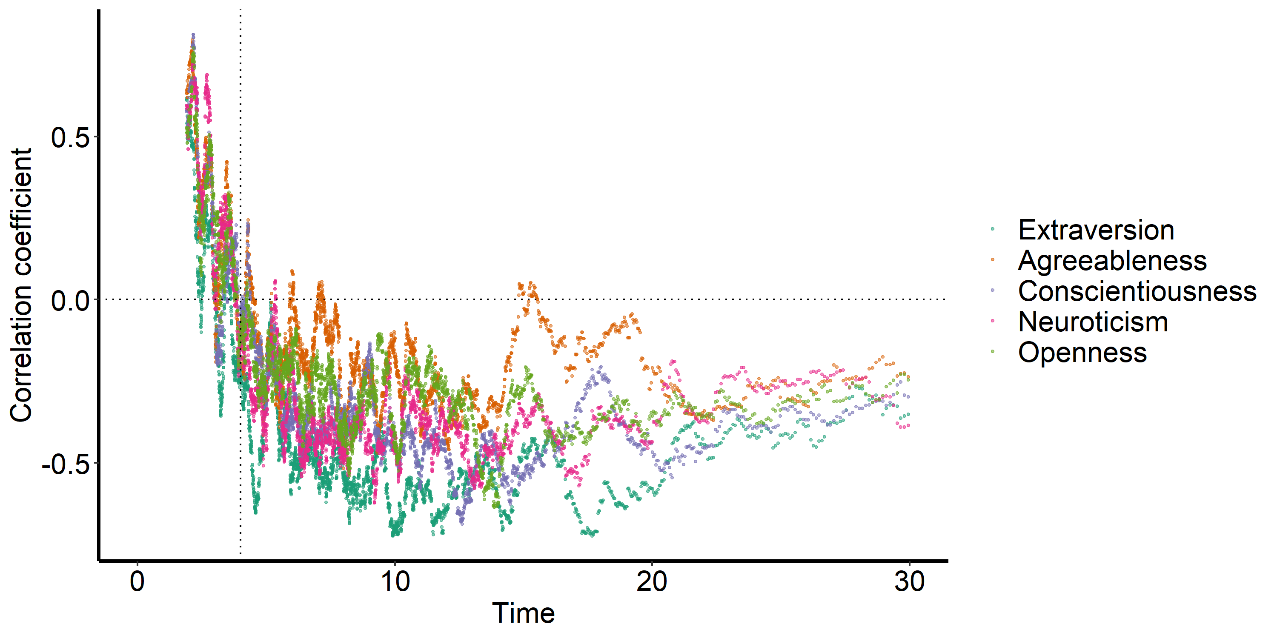


**Results**

**Hierarchical Regression Analysis**

The results of hierarchical regression analysis for the four coping-behavior factors and three risk-perception factors are displayed in Table S1–Table S7.

**Coping-behavior factors**

**Table S1.** Contributions of background factors, risk-perception factors, and psychobehavioral characteristics to CB1 (mask-wearing).

|  | **Block 1** | | | | | | | **+ Block 2** | | | | **+ Block 3** | | | |  | |
| --- | --- | --- | --- | --- | --- | --- | --- | --- | --- | --- | --- | --- | --- | --- | --- | --- | --- |
|  | B | | | SE B | β | | *f_B/A_^2^* | B | SE B | β | *f_B/A_^2^* | B | SE B | β | *f_B/A_^2^* | *∆F* |  |
| Sex | -0.667 | | | 0.051 | -0.232 | | **0.059** | -0.620 | 0.049 | -0.215 | **0.057** | -0.469 | 0.049 | -0.163 | **0.032** |  |  |
| HS_single | -0.136 | | | 0.066 | -0.037 | | 0.001 | -0.108 | 0.063 | -0.029 | 0.001 | -0.091 | 0.061 | -0.024 | 0.001 |  |  |
| Local case of infection | 0.199 | | | 0.052 | 0.067 | | 0.005 | 0.164 | 0.050 | 0.056 | 0.004 | 0.146 | 0.048 | 0.049 | 0.003 |  |  |
| High-risk age | 0.138 | | | 0.015 | 0.167 | | **0.030** | 0.099 | 0.015 | 0.120 | 0.016 | 0.047 | 0.015 | 0.057 | 0.004 |  |  |
| Knowledge | 0.084 | | | 0.019 | 0.078 | | 0.007 | 0.080 | 0.018 | 0.075 | 0.007 | 0.048 | 0.018 | 0.045 | 0.003 |  |  |
| Block 1 |  | | |  |  | | 0.106 |  |  |  |  |  |  |  |  | 61.15* |  |
| Medical concerns | | | | | |  |  | 0.101 | 0.029 | 0.068 | 0.004 | 0.175 | 0.029 | 0.118 | 0.013 |  |  |
| Shortages of daily necessities | | | | | |  |  | 0.133 | 0.024 | 0.109 | 0.010 | 0.139 | 0.024 | 0.114 | 0.012 |  |  |
| Socioeconomic concerns | | | | | |  |  | 0.287 | 0.026 | 0.209 | **0.041** | 0.185 | 0.026 | 0.135 | 0.017 |  |  |
| + Block 2 |  | |  | | |  |  |  |  |  | 0.116 |  |  |  |  | 111.06* |  |
| Altruism | |  |  | | |  |  |  |  |  |  | -0.059 | 0.028 | -0.042 | 0.002 |  |  |
| Etiquette | |  |  | | |  |  |  |  |  |  | 0.260 | 0.032 | 0.194 | **0.023** |  |  |
| Emotional regulation | | | | | |  |  |  |  |  |  | -0.064 | 0.038 | -0.043 | 0.001 |  |  |
| Self-transcendence | |  |  | | |  |  |  |  |  |  | 0.077 | 0.038 | 0.053 | 0.001 |  |  |
| Active well-being | |  |  | | |  |  |  |  |  |  | 0.078 | 0.031 | 0.058 | 0.002 |  |  |
| Agreeableness | |  |  | | |  |  |  |  |  |  | 0.082 | 0.014 | 0.107 | 0.011 |  |  |
| Openness | |  |  | | |  |  |  |  |  |  | -0.024 | 0.012 | -0.035 | 0.001 |  |  |
| + Block 3 |  | |  | | |  |  |  |  |  |  |  |  |  | 0.087 | 35.743* |  |
| *R^2^* |  | |  | | |  | 0.096 |  |  |  | 0.190 |  |  |  | 0.255 |  |  |
| *∆R^2^* |  | |  | | |  |  |  |  |  | 0.094 |  |  |  | 0.065 |  |  |

*Note:* *: *p-values* only for *∆F*, *f_B/A_^2^* > 0.02 are in bold.

**Table S2.** Contributions of background factors, risk-perception factors, and psychobehavioral characteristics to CB2 (information-seeking).

|  | **Block 1** | | | | | **+ Block 2** | | | | | **+ Block 3** | | | |  |
| --- | --- | --- | --- | --- | --- | --- | --- | --- | --- | --- | --- | --- | --- | --- | --- |
|  | B | | SE B | β | *f_B/A_^2^* | B | SE B | | β | *f_B/A_^2^* | B | SE B | β | *f_B/A_^2^* | *∆F* |
| Sex | -0.143 | | 0.042 | -0.061 | 0.004 | -0.104 | 0.038 | | -0.044 | 0.003 | -0.087 | 0.037 | -0.037 | 0.002 |  |
| Age | 0.008 | | 0.002 | 0.110 | 0.008 | 0.015 | 0.002 | | 0.210 | **0.031** | 0.008 | 0.002 | 0.116 | 0.010 |  |
| HS_single | -0.199 | | 0.054 | -0.066 | 0.005 | -0.159 | 0.050 | | -0.053 | 0.003 | -0.110 | 0.048 | -0.036 | 0.002 |  |
| Toddler | 0.150 | | 0.077 | 0.036 | 0.001 | 0.155 | 0.071 | | 0.037 | 0.002 | 0.044 | 0.068 | 0.011 | 0.000 |  |
| Local case of infection | 0.190 | | 0.042 | 0.079 | 0.007 | 0.163 | 0.039 | | 0.068 | 0.006 | 0.135 | 0.037 | 0.056 | 0.005 |  |
| High-risk age | 0.105 | | 0.015 | 0.156 | 0.016 | 0.027 | 0.015 | | 0.040 | 0.001 | 0.029 | 0.014 | 0.043 | 0.001 |  |
| Knowledge | 0.135 | | 0.016 | 0.155 | **0.026** | 0.130 | 0.014 | | 0.149 | **0.029** | 0.068 | 0.014 | 0.079 | 0.008 |  |
| Block 1 |  | |  |  | 0.119 |  |  | |  |  |  |  |  |  | 48.73* |
| Medical concerns | | |  |  |  | 0.089 | 0.024 | 0.074 | | 0.005 | 0.127 | 0.023 | 0.105 | 0.011 |  |
| Shortages of daily necessities | | | |  |  | 0.158 | 0.019 | 0.159 | | **0.023** | 0.141 | 0.018 | 0.142 | **0.021** |  |
| Socioeconomic concerns | | | |  |  | 0.264 | 0.021 | 0.237 | | **0.056** | 0.225 | 0.020 | 0.202 | **0.043** |  |
| + Block 2 |  | |  |  |  |  |  |  | | 0.175 |  |  |  |  | 167.8* |
| Leadership | |  |  |  |  |  |  |  | |  | 0.193 | 0.029 | 0.178 | 0.015 |  |
| Problem-solving | |  |  |  |  |  |  |  | |  | -0.095 | 0.032 | -0.076 | 0.003 |  |
| Altruism | |  |  |  |  |  |  |  | |  | 0.048 | 0.023 | 0.043 | 0.002 |  |
| Self-transcendence | | |  |  |  |  |  |  | |  | 0.067 | 0.029 | 0.057 | 0.002 |  |
| Active well-being | |  |  |  |  |  |  |  | |  | 0.167 | 0.025 | 0.154 | 0.016 |  |
| Extraversion | |  |  |  |  |  |  |  | |  | -0.017 | 0.009 | -0.035 | 0.001 |  |
| Conscientiousness | |  |  |  |  |  |  |  | |  | 0.028 | 0.010 | 0.052 | 0.003 |  |
| + Block 3 |  | |  |  |  |  |  |  | |  |  |  |  | 0.130 | 53.093* |
| *R^2^* |  | |  |  | 0.106 |  |  |  | | 0.239 |  |  |  | 0.327 |  |
| *∆R^2^* |  | |  |  |  |  |  |  | | 0.133 |  |  |  | 0.087 |  |

*Note:* *: *p-values* <.001 only for *∆F*, *f_B/A_^2^* > 0.02 are in bold.

**Table S3.** Contributions of background factors, risk-perception factors, and psychobehavioral characteristics to CB3 (resistance to social stagnation).

|  | | **Block 1** | | | | | **+ Block 2** | | | | **+ Block 3** | | | |  | |
| --- | --- | --- | --- | --- | --- | --- | --- | --- | --- | --- | --- | --- | --- | --- | --- | --- |
|  | | B | | SE B | β | *f_B/A_^2^* | B | SE B | β | *f_B/A_^2^* | B | SE B | β | *f_B/A_^2^* | | *∆F* |
| Sex | | -0.050 | | 0.035 | -0.026 | 0.001 | -0.025 | 0.034 | -0.013 | 0.000 | -0.139 | 0.030 | -0.074 | 0.008 | |  |
| Toddler | | 0.110 | | 0.064 | 0.033 | 0.001 | 0.084 | 0.062 | 0.025 | 0.001 | 0.020 | 0.052 | 0.006 | 0.000 | |  |
| Child | | 0.171 | | 0.057 | 0.057 | 0.003 | 0.153 | 0.056 | 0.051 | 0.003 | 0.107 | 0.047 | 0.036 | 0.002 | |  |
| Knowledge | | 0.124 | | 0.013 | 0.176 | **0.032** | 0.123 | 0.013 | 0.174 | **0.033** | 0.048 | 0.011 | 0.068 | 0.007 | |  |
| Block 1 | |  | |  |  | 0.037 |  |  |  |  |  |  |  |  | | 26.99* |
| Shortages of daily necessities | | | | |  |  | 0.161 | 0.016 | 0.200 | **0.035** | 0.143 | 0.014 | 0.178 | **0.038** | |  |
| Socioeconomic concerns | | | | |  |  | 0.065 | 0.018 | 0.072 | 0.005 | 0.099 | 0.016 | 0.110 | 0.014 | |  |
| **+** Block 2 | |  | |  |  |  |  |  |  | 0.064 |  |  |  |  | | 92.179* |
| Leadership | | |  |  |  |  |  |  |  |  | 0.422 | 0.023 | 0.483 | **0.118** | |  |
| Problem-solving | | |  |  |  |  |  |  |  |  | -0.181 | 0.029 | -0.177 | 0.014 | |  |
| Altruism | | |  |  |  |  |  |  |  |  | 0.092 | 0.019 | 0.101 | 0.009 | |  |
| Stubbornness | | |  |  |  |  |  |  |  |  | -0.051 | 0.018 | -0.052 | 0.003 | |  |
| Etiquette | | |  |  |  |  |  |  |  |  | -0.221 | 0.020 | -0.250 | **0.043** | |  |
| Emotional regulation | | | |  |  |  |  |  |  |  | 0.083 | 0.025 | 0.084 | 0.004 | |  |
| Self-transcendence | | |  |  |  |  |  |  |  |  | 0.045 | 0.024 | 0.047 | 0.001 | |  |
| Active well-being | | |  |  |  |  |  |  |  |  | 0.144 | 0.020 | 0.163 | 0.018 | |  |
| Extraversion | | |  |  |  |  |  |  |  |  | -0.018 | 0.008 | -0.047 | 0.002 | |  |
| Agreeableness | | |  |  |  |  |  |  |  |  | -0.048 | 0.009 | -0.094 | 0.009 | |  |
| Conscientiousness | | |  |  |  |  |  |  |  |  | -0.017 | 0.008 | -0.038 | 0.002 | |  |
| Openness | | |  |  |  |  |  |  |  |  | 0.046 | 0.008 | 0.103 | 0.012 | |  |
| + Block 3 |  | | |  |  |  |  |  |  |  |  |  |  | 0.429 | | 102.521* |
| *R^2^* |  | | |  |  | 0.036 |  |  |  | 0.094 |  |  |  | 0.366 | |  |
| *∆R^2^* |  | | |  |  |  |  |  |  | 0.058 |  |  |  | 0.272 | |  |

*Note:* *: *p-values* <.001 only for *∆F*, *f_B/A_^2^* > 0.02 are in bold.

**Table S4.** Contributions of background factors, risk-perception factors, and psychobehavioral characteristics to CB4 (infection-prevention).

|  | **Block1** | | | | | | **+Block2** | | | | | **+Block3** | | | |  |
| --- | --- | --- | --- | --- | --- | --- | --- | --- | --- | --- | --- | --- | --- | --- | --- | --- |
|  | B | | | SE B | β | *f_B/A_^2^* | B | SE B | β | | *f_B/A_^2^* | B | SE B | β | *f_B/A_^2^* | *∆F* |
| Sex | -0.385 | | | 0.038 | -0.179 | **0.035** | -0.361 | 0.037 | -0.167 | | **0.033** | -0.266 | 0.035 | -0.123 | **0.020** |  |
| Age | 0.006 | | | 0.002 | 0.086 | 0.005 | 0.009 | 0.002 | 0.145 | | 0.013 | -0.001 | 0.001 | -0.011 | 0.000 |  |
| Hs_single | -0.076 | | | 0.053 | -0.027 | 0.001 | -0.045 | 0.051 | -0.016 | | 0.000 | -0.059 | 0.047 | -0.021 | 0.001 |  |
| Hs_couple | 0.081 | | | 0.047 | 0.035 | 0.001 | 0.107 | 0.046 | 0.046 | | 0.002 | 0.056 | 0.041 | 0.024 | 0.001 |  |
| Toddler | 0.201 | | | 0.072 | 0.053 | 0.003 | 0.212 | 0.069 | 0.056 | | 0.003 | 0.083 | 0.063 | 0.022 | 0.001 |  |
| Local case of infection | 0.185 | | | 0.039 | 0.084 | 0.008 | 0.164 | 0.038 | 0.074 | | 0.007 | 0.127 | 0.034 | 0.057 | 0.005 |  |
| High-risk age | 0.074 | | | 0.014 | 0.120 | 0.010 | 0.027 | 0.015 | 0.044 | | 0.001 | 0.032 | 0.013 | 0.051 | 0.002 |  |
| Knowledge | 0.115 | | | 0.014 | 0.143 | **0.022** | 0.111 | 0.014 | 0.138 | | **0.022** | 0.059 | 0.013 | 0.074 | 0.007 |  |
| Block 1 |  | | |  |  | 0.119 |  |  |  | |  |  |  |  | 42.759* | |
| Medical concerns | | | | |  |  | 0.045 | 0.023 | 0.041 | | 0.002 | 0.111 | 0.021 | 0.100 | 0.010 |  |
| Shortages of daily necessities | | | | | |  | 0.076 | 0.019 | 0.084 | | 0.006 | 0.076 | 0.017 | 0.083 | 0.007 |  |
| Socioeconomic concerns | | | | |  |  | 0.195 | 0.020 | 0.190 | | **0.033** | 0.107 | 0.019 | 0.104 | 0.011 |  |
| + Block 2 |  | | |  |  |  |  |  |  | | 0.076 |  |  |  | 72.681* | |
| Altruism | | |  |  |  |  |  |  |  | |  | -0.087 | 0.021 | -0.083 | 0.006 |  |
| Stubbornness | | |  |  |  |  |  |  |  | |  | -0.068 | 0.021 | -0.061 | 0.004 |  |
| Etiquette | | |  |  |  |  |  |  |  | |  | 0.183 | 0.023 | 0.181 | **0.022** |  |
| Emotional regulation | | | | |  |  |  |  |  | |  | 0.062 | 0.027 | 0.056 | 0.002 |  |
| Self-transcendence | | | |  |  |  |  |  |  | |  | 0.045 | 0.028 | 0.042 | 0.002 |  |
| Active well-being | | | |  |  |  |  |  |  | |  | 0.192 | 0.022 | 0.191 | **0.027** |  |
| Agreeableness | | |  |  |  |  |  |  |  | |  | 0.019 | 0.011 | 0.034 | 0.002 |  |
| Conscientiousness | | | |  |  |  |  |  |  | |  | 0.067 | 0.009 | 0.135 | 0.019 |  |
| + Block 3 | |  | |  |  |  |  |  |  | |  |  |  |  | 0.222 | 79.676* |
| *R^2^* | |  | |  |  | 0.106 |  |  |  | 0.169 | |  |  |  | 0.316 |  |
| *∆R^2^* | |  | |  |  |  |  |  |  | 0.063 | |  |  |  | 0.147 |  |

*Note:* *: *p-values* only for *∆F*, *f_B/A_^2^* > 0.02 are in bold.

**Risk-perception factors**

**Table S5.** Contributions of background factors and psychobehavioral characteristics to RP1 (shortages of daily necessities).

|  | **Block 1** | | | | **+ Block 2** | | | |  |
| --- | --- | --- | --- | --- | --- | --- | --- | --- | --- |
|  | B | SE B | β | *f_B/A_^2^* | B | SE B | β | *f_B/A_^2^* | *∆F* |
| Sex | -0.114 | 0.043 | -0.048 | 0.002 | -0.069 | 0.045 | -0.029 | 0.001 |  |
| Age | -0.016 | 0.002 | -0.225 | **0.032** | -0.013 | 0.002 | -0.176 | 0.017 |  |
| Child | 0.159 | 0.069 | 0.042 | 0.002 | 0.159 | 0.067 | 0.043 | 0.002 |  |
| High-risk age | 0.101 | 0.018 | 0.149 | 0.011 | 0.080 | 0.017 | 0.119 | 0.007 |  |
| High-risk family member | 0.054 | 0.013 | 0.089 | 0.006 | 0.044 | 0.012 | 0.073 | 0.004 |  |
| Block 1 |  |  |  | 0.052 |  |  |  |  | 30.109* |
| Problem-solving |  |  |  |  | 0.102 | 0.039 | 0.080 | 0.002 |  |
| Altruism |  |  |  |  | 0.060 | 0.027 | 0.053 | 0.002 |  |
| Stubbornness |  |  |  |  | 0.044 | 0.027 | 0.036 | 0.001 |  |
| Etiquette |  |  |  |  | 0.106 | 0.030 | 0.096 | 0.004 |  |
| Self-transcendence | |  |  |  | 0.049 | 0.033 | 0.042 | 0.001 |  |
| Extraversion |  |  |  |  | -0.034 | 0.010 | -0.069 | 0.004 |  |
| Agreeableness |  |  |  |  | -0.030 | 0.014 | -0.048 | 0.002 |  |
| Conscientiousness | |  |  |  | -0.046 | 0.012 | -0.085 | 0.005 |  |
| Neuroticism |  |  |  |  | 0.078 | 0.013 | 0.141 | 0.014 |  |
| Openness |  |  |  |  | 0.018 | 0.012 | 0.032 | 0.001 |  |
| + Block 2 |  |  |  |  |  |  |  | 0.060 | 17.261* |
| *R^2^* | 0.050 |  |  |  | 0.104 |  |  |  |  |
| *∆R^2^* |  |  |  |  | 0.054 |  |  |  |  |

*Note:* *: *p-values* only for *∆F*, *f_B/A_^2^* > 0.02 are in bold.

**Table S6.** Contributions of background factors and psychobehavioral characteristics to RP2 (medical concerns).

|  | **Block 1** | | | | | **+ Block 2** | | | |  |
| --- | --- | --- | --- | --- | --- | --- | --- | --- | --- | --- |
|  | B | | SE B | β | *f_B/A_^2^* | B | SE B | β | *f_B/A_^2^* | *∆F* |
| Age | -0.017 | | 0.001 | -0.296 | **0.061** | -0.012 | 0.001 | -0.201 | **0.026** |  |
| Hs_twogener | 0.078 | | 0.035 | 0.040 | 0.002 | 0.081 | 0.034 | 0.041 | 0.002 |  |
| Child | 0.091 | | 0.054 | 0.030 | 0.001 | 0.106 | 0.052 | 0.035 | 0.001 |  |
| Local case of infection | 0.078 | | 0.033 | 0.039 | 0.002 | 0.066 | 0.032 | 0.033 | 0.001 |  |
| Chronic disease | 0.053 | | 0.013 | 0.088 | 0.006 | 0.050 | 0.013 | 0.082 | 0.005 |  |
| High-risk age | 0.196 | | 0.016 | 0.351 | **0.054** | 0.184 | 0.015 | 0.330 | **0.050** |  |
| High-risk family member | 0.089 | | 0.010 | 0.177 | **0.029** | 0.079 | 0.009 | 0.158 | **0.024** |  |
| Block 1 |  | |  |  | 0.249 |  |  |  |  | 102.241* |
| Problem-solving | |  |  |  |  | 0.179 | 0.030 | 0.171 | 0.013 |  |
| Altruism | |  |  |  |  | 0.056 | 0.019 | 0.060 | 0.003 |  |
| Etiquette | |  |  |  |  | 0.042 | 0.021 | 0.046 | 0.001 |  |
| Emotional regulation | |  |  |  |  | -0.062 | 0.027 | -0.061 | 0.002 |  |
| Extraversion | |  |  |  |  | -0.030 | 0.007 | -0.076 | 0.006 |  |
| Agreeableness | |  |  |  |  | -0.052 | 0.010 | -0.100 | 0.009 |  |
| Conscientiousness | |  |  |  |  | -0.049 | 0.009 | -0.110 | 0.010 |  |
| Neuroticism | |  |  |  |  | 0.058 | 0.010 | 0.128 | 0.013 |  |
| + Block 2 |  | |  |  |  |  |  |  | 0.074 | 26.601* |
| *R^2^* |  | |  |  | 0.199 |  |  |  | 0.255 |  |
| *∆R^2^* |  | |  |  |  |  |  |  | 0.055 |  |

*Note:* *: *p-values* only for *∆F*, *f_B/A_^2^* > 0.02 are in bold.

**Table S7.** Contributions of background factors and psychobehavioral characteristics to RP3 (socioeconomic concerns).

|  | **Block 1** | | | | **+ Block 2** | | | |  |
| --- | --- | --- | --- | --- | --- | --- | --- | --- | --- |
|  | B | SE B | β | *f_B/A_^2^* | B | SE B | β | *f_B/A_^2^* | *∆F* |
| Sex | -0.064 | 0.039 | -0.031 | 0.001 | 0.052 | 0.039 | 0.025 | 0.001 |  |
| Age | -0.009 | 0.002 | -0.138 | 0.011 | -0.008 | 0.002 | -0.134 | 0.010 |  |
| Local case of infection | 0.103 | 0.040 | 0.048 | 0.002 | 0.076 | 0.037 | 0.035 | 0.001 |  |
| Chronic disease | -0.037 | 0.015 | -0.056 | 0.002 | -0.029 | 0.015 | -0.044 | 0.001 |  |
| High-risk age | 0.118 | 0.019 | 0.197 | 0.014 | 0.093 | 0.018 | 0.154 | 0.010 |  |
| High-risk family member | 0.040 | 0.011 | 0.073 | 0.004 | 0.035 | 0.011 | 0.065 | 0.004 |  |
| Knowledge | 0.022 | 0.015 | 0.028 | 0.001 | -0.003 | 0.014 | -0.004 | 0.000 |  |
| Block 1 |  |  |  | 0.037 |  |  |  |  | 15.028* |
| Leadership |  |  |  |  | -0.099 | 0.023 | -0.102 | 0.006 |  |
| Problem-solving |  |  |  |  | 0.184 | 0.034 | 0.163 | 0.010 |  |
| Stubbornness |  |  |  |  | 0.060 | 0.023 | 0.055 | 0.002 |  |
| Etiquette |  |  |  |  | 0.209 | 0.026 | 0.213 | **0.023** |  |
| Self-transcendence |  |  |  |  | 0.060 | 0.028 | 0.057 | 0.002 |  |
| Agreeableness |  |  |  |  | 0.030 | 0.012 | 0.053 | 0.002 |  |
| Conscientiousness |  |  |  |  | -0.042 | 0.010 | -0.087 | 0.006 |  |
| Neuroticism |  |  |  |  | 0.073 | 0.010 | 0.149 | 0.017 |  |
| + Block 2 |  |  |  |  |  |  |  | 0.128 | 45.795* |
| *R^2^* |  |  |  | 0.035 |  |  |  | 0.145 |  |
| *∆R^2^* |  |  |  |  |  |  |  | 0.109 |  |

*Note:* *: *p-values* only for *∆F*, *f_B/A_^2^* > 0.02 are in bold.

References:

1. Chmielewski M, Kucker SC. An MTurk crisis? Shifts in data quality and the impact on study results. *Soc Psychol Personal Sci* (2020) 11:464–473. doi: 10.1177/1948550619875149
2. Wood D, Harms PD, Lowman GH, DeSimone JA. Response speed and response consistency as mutually validating indicators of data quality in online samples. *Soc Psychol Personal Sci* (2017) 8:454–464. doi: 10.1177/1948550617703168
